# Stay away from me: Coughing increases social distance even in a virtual environment

**DOI:** 10.1371/journal.pone.0279717

**Published:** 2022-12-28

**Authors:** Masahiro Shiomi, Atsumu Kubota, Mitsuhiko Kimoto, Takamasa Iio, Katsunori Shimohara

**Affiliations:** 1 Department of Agent Interaction Design Laboratory, Advanced Telecommunications Research Institute International, Kyoto, Japan; 2 Faculty of Science and Engineering, Doshisha University, Kyoto, Japan; 3 Faculty of Culture and Information Science, Doshisha University, Kyoto, Japan; Tsinghua University, CHINA

## Abstract

This study investigated whether the coughing behaviors of virtual agents encourage infection avoidance behavior, i.e., distancing behaviors. We hypothesized that the changes in people’s lifestyles in physical environments due to COVID-19 probably influence their behaviors, even in virtual environments where no infection risk is present. We focused on different types of virtual agents because non-human agents, such as robot-like agents, cannot spread a virus by coughing. We prepared four kinds of virtual agents (human-like/robot-like and male/female) and coughing behaviors for them and experimentally measured the personal distance maintained by participants toward them. Our experiment results showed that participants chose a greater distance from coughing agents, regardless of the types, and negatively evaluated them. They also chose a greater distance from male agents than from female agents.

## Introduction

Understanding the perception differences between physical and virtual environments is essential for designing the behaviors of virtual agents. For example, people usually maintain a certain distance during interactions with others in physical environments, an idea called proxemics [1, 2]. Several studies investigated whether it can be applied to interactions in human-computer interaction contexts. For example, one study reported that human perception of distance does not change much between physical and virtual environments [3]. Other studies suggested that one’s preferred distance from virtual agents is affected by complex relationships among the genders of both the users and agents as well as friendliness toward them [4, 5]. These studies suggested that addressing such knowledge in physical environments is critical for designing acceptable virtual agents in virtual environments.

If lifestyles in physical environments influence interaction styles in virtual environments, shouldn’t we also consider how the COVID-19 pandemic has changed lifestyles in virtual environments? Obviously, it has dramatically affected lifestyles related to such infection avoidance behaviors as social distancing, wearing a mask, cough etiquette, etc. In fact, recent studies have described the changes in social distancing and sociable space fueled by COVID-19 [6, 7]. Other studies reported the changes in personal spaces in post-COVID-19 situations in both physical and virtual environments, although these studies offered conflicting results: personal spaces increased even in virtual environments [8]; people share a closer distance in a virtual environment than in a physical environment [9].

**Fig 1:**
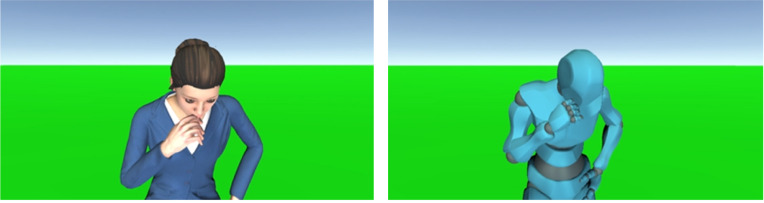
Coughing behaviors of virtual agents. Left: human-like agent, right: robot-like agent.

Although the COVID-19 pandemic is accelerating the need for virtual agents that provide services in virtual environments to avoid the risk of infections, these contrary findings complicate the behavior design guidelines of virtual agents during this pandemic. Clarifying such guidelines based on accumulating psychological knowledge about human behaviors in a virtual environment will contribute to achieving acceptable agent behaviors. For this purpose, observing people’s reactions toward such specific behaviors (as coughing) that remind them of the virus’s spread is effective in identifying why personal distance is changing in virtual environments (Fig 1, left). If an agent’s coughing behavior does not influence people’s distancing behaviors, such a result implies that people are adopting different interaction styles in a virtual environment. On the other hand, if people simply continue to maintain distance from a coughing agent, even in a virtual environment, such phenomena also provide useful knowledge for designing guidelines for virtual agent behaviors, e.g., whether virus-spreading behaviors should be prohibited in virtual environments. Based on these considerations, we address the following research question:

Research question 1 (RQ1): Do coughing virtual agents encourage social distancing behaviors?

This study also focuses on the appearance of coughing virtual agents. Past studies concluded that several factors related to the appearances of virtual agents (e.g., face design, gender, numbers, and rendering approach, e.g., realistic or a toon-CG style) influence the distancing behaviors of people [10–13]. These studies concluded that the appearance of agents is one essential factor in the distancing behaviors of people in virtual environments. We believe that when the agents are not human, e.g., a humanoid robot (Fig 1, right), people’s distancing behavior will be mitigated because a coughing robot cannot spread a virus. Based on these considerations, we pose another research question:

Research question 2 (RQ2): Do the types of agents in virtual environments change distancing behavior?

## Related works

### Distancing in a virtual environment

People establish distance in conversational interactions in both virtual and physical environments [3]. Therefore, researchers have implemented behaviors to maintain certain distances between users depending on the situation to achieve acceptable conversational interaction in virtual environments [14, 15]. Similar to physical environments, people’s personal space preferences are influenced by several factors, e.g., the agents’ emotional expressions [16] and touching behaviors [17, 18]. These studies suggested that addressing such knowledge is critical for designing acceptable virtual agents in a virtual environment.

Lifestyles also influence people’s distancing behaviors in physical environments. A recent social psychology study reported how personal space has grown in post-COVID-19 situations in both physical and virtual environments where no infection of risk is present [8]. This study argued that the personal space changes in both physical and virtual environments are significantly correlated with attitudes toward COVID-19. Another recent study concluded that the personal distance measured in a virtual environment is larger than in a past study [10]. On the other hand, another study reported that people share a closer distance in a virtual environment than in a physical environment regardless of the density of the crowd [9]. This research concluded that the presence of other virtual agents around users did not influence their social distancing.

Such conflicting social psychology reports suggest that people are modifying their lifestyles in physical environments to virtual environments or adopting different styles because behavior to avoid infection is unnecessary. To clarify people’s perception of distancing in virtual environments, in this study, we investigated the effects of coughing behaviors in a virtual environment where obviously no risk of infection exists, even though such behaviors are strongly related to infection in our physical environment. Focusing on coughing behaviors is one unique point of our study compared to past similar studies that investigated people’s perception of distancing in virtual environments.

### Appearance of virtual agents

One essential factor of distancing in virtual environments is the appearance of virtual agents. Past studies concluded that several factors related to the appearances of virtual agents (e.g., face design, gender, numbers, motions, and rendering approach, e.g., realistic or a toon-CG style) influence the distancing behaviors of people [5, 10–13, 19]. For example, Zibrek et al. reported that attractive motions reduced personal distances in a virtual environment regardless of the genders of the participants [5]. Lisi et al. experimentally compared male- and female-appearing avatars with heterosexual and non-heterosexual participants and reported that implicit sexual prejudice might increase personal distance [11]. From another perspective, researchers investigated the effects of a robot’s appearance and gender on personal space. Li et al. investigated the differences of personal distances between physical and virtual environments with a Pepper robot and concluded that personal distance in the former is smaller than in the latter, even though the appearances of the robots in both environments were identical [20]. These studies showed how people change their personal distances due to the characteristics of virtual agents.

However, these studies less focused on the relationships between infection-related behaviors and the appearances of agents. Although a past study investigated the effects of mask-wearing virtual agents [21], it only used human-appearance virtual agents. Thus, when there is no risk of infection, it remains unknown how people in a virtual environment will react to a virtual agent with a non-human-like appearance (i.e., robot-like) and whether they will keep a shorter distance in the context of infection avoidance. A unique point of our study is that we compared the relationships between agents’ appearances and distancing through coughing behaviors.

## Experiment design

### Task design

**Fig 2:**
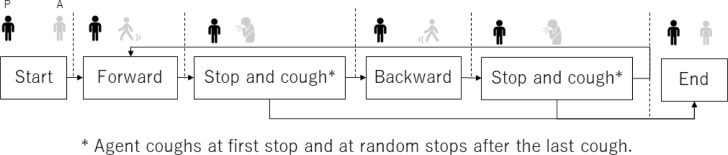
Summary of agents’ actions. Participants can control forward/backward behaviors until choosing a distance.

In this study, we followed a traditional task design to measure comfortable distance [20, 22]. Participants manually controlled the positions of virtual agents to change the distances between them. Similar to these past studies, the virtual agents are in front of the participants who only controlled their forward/backward motions. To answer RQ1, we added a coughing behavior to the virtual agents. The agent coughed when it was stopped by the participants (Fig 2). We designed the coughing timing for beginning at the first stop timing and at a random stop timing after the last cough to avoid getting used to agents’ coughing.

### Appearance and coughing design

**Fig 3:**
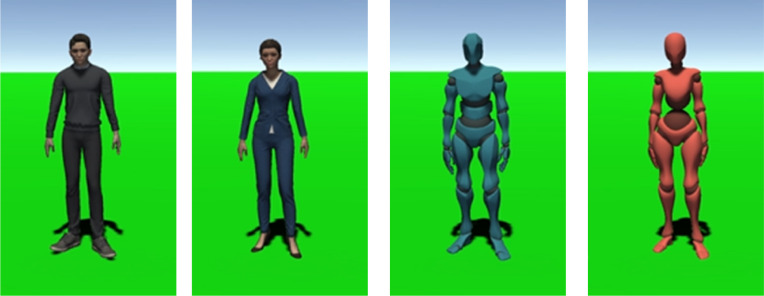
Appearance of each virtual agent. We used four different agents in the experiment. (a) human-like man, (b) human-like woman, (c) robot-like man, (d) robot-like woman.

In this study, to answer RQ2, we prepared four virtual agents based on two design factors: type (human-like and robot-like), and gender (male and female) (Fig 3), because robot-like agents might be perceived as being unable to spread the virus even though they are coughing. The heights of the agents were similar (165 ~ 170 cm) to avoid any bias caused by height differences.

For the coughing behavior, we used a cough motion where the agents looked down and put a clenched right fist over their mouths (Fig 1). We also played a coughing sound during the motion. While the agents were coughing, they randomly repeated coughing motions (one to three times) and sounds.

## Experiment

### Hypotheses and predictions

The COVID-19 pandemic is changing our lifestyles, especially distancing behaviors [6, 7]. Several studies have reported that people’s distancing behaviors have even changed in virtual environments where no infection risks are possible [8, 21], suggesting that the changes of people’s lifestyles in physical environments influence their behaviors in virtual environments. Since coughing is a typical way to spread a virus by expelling germs into the environment [23, 24], people might choose to maintain a distance from those who are coughing. Based on these considerations, people in a virtual environment also maintain a distance from coughing virtual agents. Therefore, we made the following hypothesis about distancing behavior:

**Prediction 1:** People will maintain greater distance from virtual agents that are coughing and positively evaluate those that are not coughing.

An agent’s appearance is another factor in distancing behavior in virtual environments [5, 10–13]. In the context of viral spread, such artificial objects as a robot will be perceived as a safe interaction target because robots cannot be infected by a virus. In fact, several robots have been used in our environments to provide services without infection risks [25, 26]. A past study reported no significant difference in preferred personal distances between human-like and robot-like agents in a virtual environment [19]. However, if people’s lifestyles in their physical environments influence their behaviors in virtual environments, they will maintain less distance in robot-like virtual environments. Therefore, we made the following hypothesis about distancing behavior:

**Prediction 2:** People will maintain less distance from robot-like agents than human-like agents, i.e., a weaker coughing behavior effect in the robot-like agents than in the human-like agents.

### Environment

We conducted our experiment at our laboratory. The participants used Oculus Rift S and hand controllers and joined our virtual environment, implemented by Unity. The virtual agent was initially placed rather far (about seven meters) from the participant.

### Participants

Thirty participants (15 women and 15 men, average age: 21.93, S.D.: 1.82) joined our experiment.

### Conditions: Type, appearance, and coughing

We prepared three factors: type (*human-like*/*robot-like*), gender (*male*/*female*), and coughing (*with-coughing*/*without-coughing*) (Fig 2). We employed a within-subject design in which each participant was randomly assigned to all eight conditions considering counterbalances. We focused on the agents’ genders because several studies in the early stage of proxemics research concluded that the effects of participant genders are insignificant when participants are approached by another person [27–31]. On the other hand, some studies reported that the gender of the approacher causes significant effects in identical situations [29–31], e.g., participants maintained more distance from males than females. Based on these considerations and to avoid having excessive factors in our analysis, we focused only on the agent’s genders in this study. We also did not evaluate the age effects because a past study reported that there are no differences in personal space between older and younger adults [32].

**Type factor:** In the *human-like* condition, the participants meet *human-like* male or female agents (i.e., depending on the *gender* factor) in the virtual environment (Fig 3A and 3B). In the *robot-like* condition, the participants meet *robot-like* male or female agents in the virtual environment (Fig 3C and 3D).

**Gender factor:** In the *male* condition, the participants meet *human-like* or *robot-like* male agents (i.e., depending on the *type* factor) in the virtual environment (Fig 3A and 3C). In the *female* condition, the participants meet *human-like* or *robot-like* female agents in the virtual environment (Fig 3B and 3D).

**Coughing factor:** In the *with-coughing* condition, the virtual agent randomly made coughing motions (one to three times) during the coughing behaviors and a coughing sound (see Fig 2). The virtual agent never coughed in the *without-coughing* condition.

### Measurement

We measured two subjective items to investigate how coughing agents influence distancing behaviors: the distance from each virtual agent (the center points of the participants and virtual human’s heads) and the avoidance ratios after coughing. In addition, we investigated their perceived feelings toward the virtual agents with two Godspeed questionnaire scales [33], which is one of the commonly used questionnaires in human-robot interaction studies. We employed five items from the likeability scale (dislike-like, unfriendly-friendly, unkind-kind, unpleasant-pleasant, and awful-nice) and three from the safety scale (anxious-relaxed, agitated-calm, and quiescent-surprised). The items were assessed on a 1-to-7 response format for the pairs of adjectives.

### Procedure

All the procedures were approved by the Advanced Telecommunication Research Review Boards (21-501-3). After the participants provided written, informed consent, the researcher clearly explained the experiment’s procedure and its aim, e.g., measuring their preferred distance. The participants read explanations of the experiment and how to control the agents in the virtual environment. Similar to past proxemics studies on comfortable distance, participants controlled the positions of the virtual agents to investigate their preferred distance in a virtual environment. Our participants stood in the physical environment, stopped, and fixed their position in the virtual environment. Therefore, they did not move during the experiments. They manipulated the agents’ forward/backward behaviors by hand controllers and chose their preferred distance. After finalizing the positions of each virtual agent, they answered questionnaires.

## Results

### Distance with virtual agents

**Fig 4:**
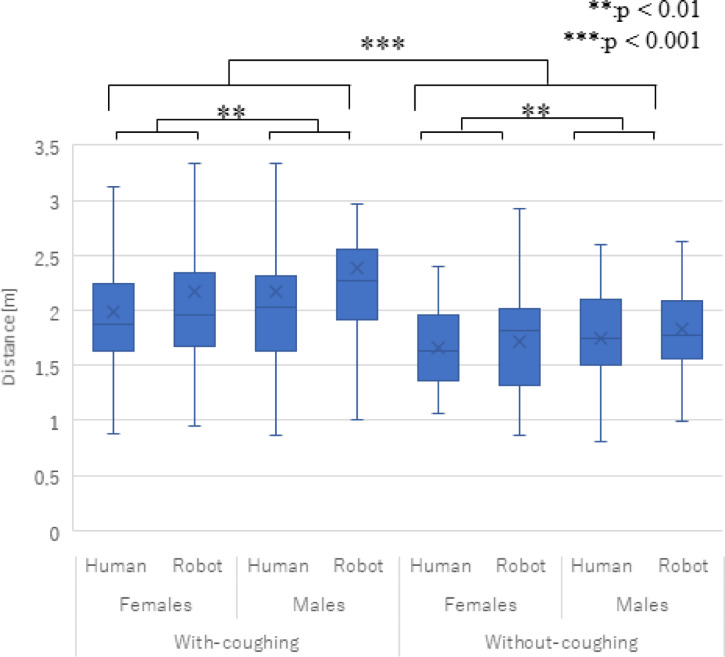
Average and S.E. of distance from virtual agents. The *gender* and *coughing* factors showed significant main effects.

Fig 4 shows the distances toward the virtual agents (average and standard error (S.E.)). We conducted a three-factor ANOVA for the *type*, *gender*, and *coughing* factors and identified significant main effects in the gender (*F*(1,29) = 11.990, *p =* 0.002, *partial η2* = 0.293) and coughing factors (*F*(1,29) = 13.392, *p <* 0.001, *partial η2* = 0.316).

We did not find any significant effects in the *type* factor (*F*(1,29) = 3.066, *p =* 0.090, *partial η2* = 0.096), the two-way interaction effects between the *type* and *gender* factors (*F*(1,29) = 0.188, *p =* 0.668, *partial η2* = 0.006), the two-way interaction effects between the *type* and *coughing* factors (*F*(1,29) = 2.609, *p =* 0.117, *partial η2* = 0.083), the two-way interaction effects between the *gender* and *coughing* factors (*F*(1,29) = 1.531, *p =* 0.226, *partial η2* = 0.050), or in the three-way interaction effects among the factors (*F*(1,29) = 0.013, *p =* 0.909, *partial η2* = 0.001).

### Avoidance after coughing

**Fig 5:**
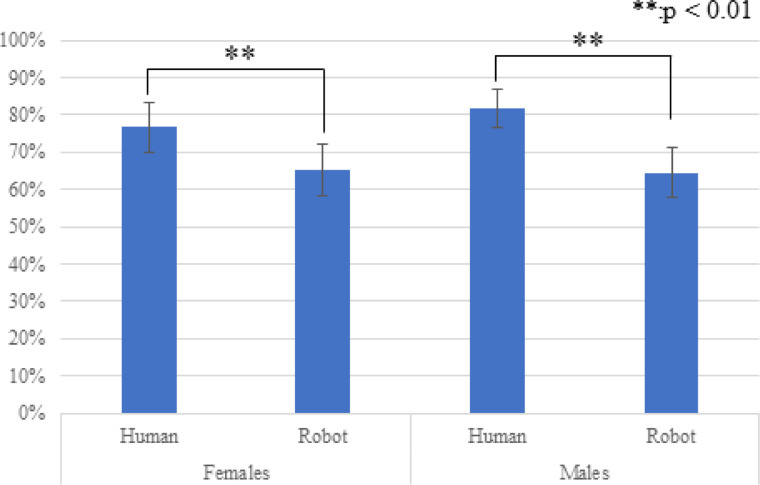
Average and S.E of avoidance ratios after coughing. The *type* factor showed a significant main effect.

Fig 5 shows the avoidance ratios after the coughing of the virtual agents (average and S.E.). Since this analysis was conducted only in the *with-coughing* condition, we conducted a two-factor ANOVA for the type and gender factors and identified a significant main effect in the *type* factor (*F*(1,29) = 5.951, *p =* 0.021, *partial η2* = 0.170). We did not find any significant effects in the *gender* factor (*F*(1,29) = 0.150, *p =* 0.702, *partial η2* = 0.005) or in the interaction effects (*F*(1,29) = 0.314, *p =* 0.580, *partial η2* = 0.011).

### Questionnaire results

**Fig 6:**
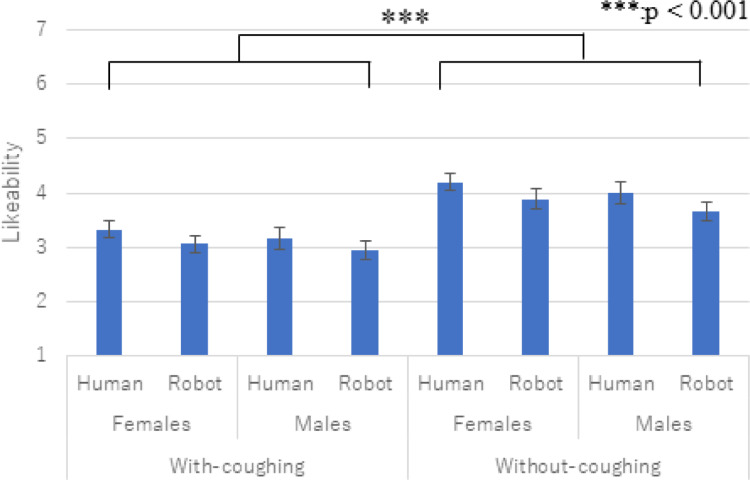
Average and S.E of likeability. The *coughing* factor showed a significant main effect.

Fig 6 shows the questionnaire results of likeability. We conducted a three-factor ANOVA and identified a significant main effect only in the *coughing* factor (*F*(1,29) = 35.736, *p <* 0.001, *partial η2* = 0.552).

We did not find any significant effects in the *type* factor (*F*(1,29) = 3.326, *p =* 0.079, *partial η2* = 0.103), the gender factor (*F*(1,29) = 1.911, *p =* 0.177, *partial η2* = 0.062), or in the two-way interaction effects between the *type* and *gender* factors (*F*(1,29) = 0.002, *p =* 0.961, *partial η2* = 0.001), the two-way interaction effects between the *type* and *coughing* factors (*F*(1,29) = 0.376, *p =* 0.544, *partial η2* = 0.013), the two-way interaction effects between the *gender* and *coughing* factors (*F*(1,29) = 0.547, *p =* 0.465, *partial η2* = 0.019), or the three-way interaction effects among the factors (*F*(1,29) = 0.124, *p =* 0.727, *partial η2* = 0.004).

**Fig 7:**
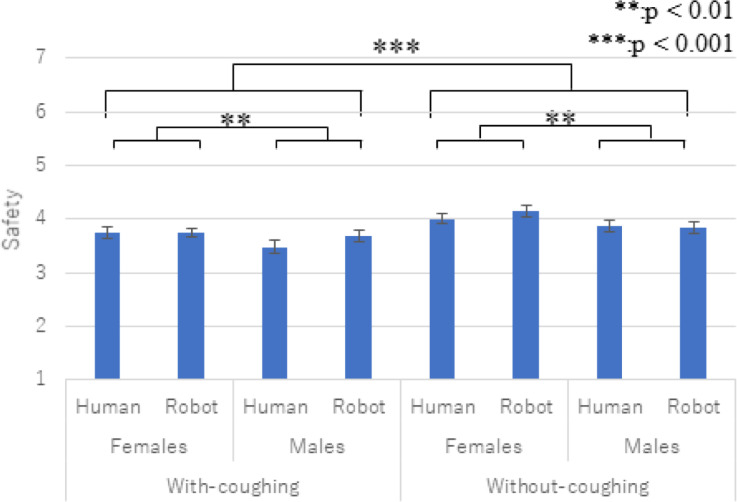
Average and S.E of safety. The *gender* and *coughing* factors showed significant main effects.

Fig 7 shows the questionnaire results for safety. We conducted a three-factor ANOVA and identified significant main effects in the *gender* (*F*(1,29) = 5.965, *p =* 0.021, *partial η2* = 0.021) and *coughing* factors (*F*(1,29) = 25.657, *p <* 0.001, *partial η2* = 0.469).

We did not find any significant effects in the *type* factor (*F*(1,29) = 0.703, *p =* 0.409, *partial η2* = 0.024) or in the two-way interaction effects between the *type* and *gender* factors (*F*(1,29) = 0.002, *p =* 0.964, *partial η2* = 0.001), the two-way interaction effects between the *type* and *coughing* factors (*F*(1,29) = 0.262, *p =* 0.612, *partial η2* = 0.009), the two-way interaction effects between the *gender* and *coughing* factors (*F*(1,29) = 0.323, *p =* 0.574, *partial η2* = 0.011), or the three-way interaction effects among the factors (*F*(1,29) = 2.715, *p =* 0.110, *partial η2* = 0.086).

### Summary and verifications of predictions

Regarding the coughing effects, our experiment results showed that the participants maintained more distance from the virtual agents when they were coughing. In addition, both questionnaire results showed that the participants positively evaluated the virtual agents when they were not coughing. Thus, prediction 1 is supported and answers research question 1: coughing virtual agents encourage distancing behaviors.

Regarding the appearance effects, the experiment results did not show distance differences between the human-like and robot-like agents, although the participants showed more distancing reactions toward the human-like agents than robot-like agents after their coughing behaviors. Thus, prediction 2 is partially supported, and answers research question 2: coughing from human-like virtual agents caused more distancing reactions by people.

## Discussion

### People’s reaction toward coughing agents in virtual environments

Our experiment results resembled those of past work that argued that changes in personal spaces increased even in virtual environments [8, 10], even though coughing by agents poses absolutely no infection risk. The questionnaire results also showed negative feelings toward coughing agents. Moreover, the participants avoided the human-like agents more than the robot-like agents after the former coughed. About RQ1, we thought that the changes in lifestyle during the COVID-19 pandemic would have influences in the context of social anxiety. A past study reported that social anxiety influences distancing behaviors even in a virtual environment [34]. Coughing voices in a virtual environment may cause social anxiety in the participants due to their lifestyles during the COVID-19 pandemic. It also may make people keep a greater distance from coughing agents.

About RQ2, we observed negative attitudes toward robot-like agents that cough, although they cannot pose any threat of spreading a virus. This result suggests that participants anthropomorphized the robot-like agents, e.g., regarding coughing as risky behavior. Although the agents’ appearances were not human-like (such as animals or creatures), these phenomena may occur. Moreover, the avoidance ratios after coughing the robot-like agents were significantly lower than the human-like agents. This phenomenon might indicate that the amount of movement per distancing reaction may be different between human-like and robot-like agents. We thought that the number of distancing reactions would be related to the risk of infection. In fact, the participants felt more realistic negative feelings toward coughing by human-like agents than robot-like agents.

Although the type factor did not show significant difference, the *p*-value indicates a significant trend (*p* = 0.090), therefore, an additional discussion would be needed. One possible reason is perceived likeability. The statistical analysis showed a significant trend in the likeability score similar to the distance measurements regardless of the coughing behavior; therefore, participants’ perceived likeability may influence their distancing behaviors.

One technical challenge to solving this negative impression of coughing is to achieve a function that automatically cancels coughing behaviors. Such a function would be useful when people control their agents, e.g., virtual YouTube delivery events. Since several studies proposed methods that detect coughing sounds, implementing them will fuel the development of such functions [35–37]. An autonomous-behavior generation function that reflects coughing etiquette, e.g., covering one’s mouth/nose with a tissue/elbow, also mitigates negative impressions toward immediate coughing behaviors. Placing a mask on the agent’s mouth is also effective [21].

### Design implications for physical environment

This study provides several implications about personal distance. First, our experiment results show that the agents’ gender influenced personal distance, like in past studies [29–31] on the relationships between gender and personal distance in physical environments. Our study suggests that the gender of the approaching target has similar effects on personal distance regardless of the particular environment. This knowledge might be useful for determining conversational distances not only for autonomous virtual agents who approach users but also for such physical agents as robots.

Although we only experimented in virtual environments, our results can be used for a real-life scenario in the context of a teleoperated robot. Robots are being used more often worldwide to achieve high-quality conversational services and avoid infection risks [25, 26], and human operators may support such robots for safety and security reasons. However, if an operator coughs during interaction services, users might experience negative impressions, as in our experiments. In such a situation, as described in the above section, an autonomous-canceling function for coughing behaviors would be useful for teleoperated robots when an operator coughs.

### Gender effects

Our experiment results showed that participants kept more distance from male agents than female agents in the virtual environment. Previous studies that investigated personal distance in the physical environment reported that participants maintained more distance from males than females [29–31]. Therefore, since the gender of human beings itself has been shown to make a difference in personal distance, we thought that the gender of the virtual agents affected participants’ personal distance.

In this study, we focused only on the agent’s genders in this study to avoid having excessive factors in our analysis. As a reference, we additionally evaluate the effects of the participants’ gender toward the distance, i.e., we conducted a four-factor ANOVA for the type, agents’ gender, participants’ gender and coughing factors to investigate the effects of the combinations between agents’ genders and participants’ genders.

The results did not show any significant differences related to the participants’ gender factors, including the interaction effects (participants’ gender factor: p = 0.360, agents’ gender and participants’ gender factors: p = 0.167, type and participants’ gender factors: p = 0.914, coughing and participants’ gender factors: p = 0.130, agents’ gender, type and participants’ gender factors: p = 0.679, agents’ gender, coughing and participants’ gender factors: p = 0.264, type, coughing and participants’ gender factors: p = 0.275, type, agents’ gender, coughing and participants’ gender factors: p = 0.445). Therefore, the participants’ genders are insignificant as well as the reported results from the past studies [27–31].

### Future work and limitations

Although our study provided useful knowledge, it suffers from several limitations, including using specific virtual agents and specific virus-spreading behaviors. Our future work will investigate the influence of different kinds of agents and behaviors, e.g., animals or pet-like agents and sneezing or wearing a mask. Since we only conducted our experiment with Japanese participants, we must consider generality, cultural differences, and vaccination history. From another perspective, the relationships between users who control virtual agents also influences personal distance. For example, even though an agent is coughing, the personal distance might not increase if a friend or a family member is controlling it.

In this study, we conducted experiments with relatively young participants and adult-appearance virtual agents. Attitudes toward COVID-19 and the perceived infection risk undoubtedly differ by age [38, 39]. Therefore, future investigations of coughing effects in virtual environments with different age groups of participants and virtual agents will be useful for understanding the effects of age. Senior citizens might maintain more distance from coughing agents due to higher ratios of death from COVID in their cohort. The body size of virtual agents is another influence on distancing behaviors. Past studies concluded that personal distance increases due to the height, arm length, and face size of interaction targets (e.g., persons, virtual agents, and robots) [40–44]. Therefore, we assume that such coughing behavior of a larger agent would strongly influence distancing behaviors.

We only measured the distance between the participants and virtual agents in face-to-face situations. Therefore, investigating the shape of personal spaces around the participants is another possible future focus to understand the position relationships between virtual agents that cough. In fact, several studies on proxemics investigated the shape of personal spaces and reported differences between the front and other directions for interaction targets [2, 44, 45]. Investigating the body directions of the participants, e.g., whether they turn their body away from the agents when they cough, would also be useful to understand human reaction behaviors.

One future work will investigate how coughing affects distancing behavior in both physical and virtual environments post COVID-19. Such a comparison will elucidate how lifestyle changes in physical environments influence behaviors in virtual environments. Investigating whether cough etiquette behaviors mitigate negative impressions and distancing behaviors in virtual environments will also provide useful knowledge for the behavior designs of virtual agents.

We note that our experiment setting is relatively simple; for example, investigating complex behaviors such as approaching from different angles, shapes of personal spaces, and trajectory changes of avoiding behaviors would be additional promising future works of this study. However, the main aim of this study is to investigate the coughing effects and agents’ appearance effects in the virtual environment, which is not investigated yet in past studies. For this purpose, we believe that following the simple and basic experiment procedure would be useful to contribute to the accumulation of knowledge. Therefore, we followed existing studies’ protocols to investigate the personal distance from virtual agents in the virtual environment. It enables other researchers to follow our experiment protocols and extend them easily. Moreover, our experiment results can be used as a reference when they investigate different factors and/or complex situations.

## Conclusion

This study investigated the effects of coughing agents and their appearances (types and genders) on the distancing behaviors of participants in a virtual environment. We prepared different kinds of virtual agents and conducted an experiment where agents coughed while we measured their personal distances. Our experiment results showed that coughing caused participants to lengthen their own personal distance regardless of the type of agents, even in environments without any actual risk of infection. Moreover, when they coughed, the participants showed more distancing behaviors toward human-like agents than robot-like agents. The participants also maintained a greater distance from male agents than female agents, similar to past studies in physical environments.

These results suggest that people’s behaviors in virtual environments are affected by their lifestyles in their physical environments. This knowledge will contribute to the designs of agents’ behaviors, including autonomous virtual agents and teleoperated agents such as virtual avatars and teleoperated robots. In this context, we conclude that removing the sound of a cough and creating an autonomous function that generates cough etiquette behavior will mitigate negative impressions toward coughing behaviors, particularly when people are controlling virtual agents.

## Supporting information

S1 File Anonymized data set. (XLSX)

## References

[1] Hall E. T., and Hall E. T., *The hidden dimension*: Anchor, 1966.

[2] Hecht H., Welsch R., Viehoff J., and Longo M. R., “The shape of personal space,” *Acta psychologica*, vol. 193, pp. 113–122, 2019. doi: 10.1016/j.actpsy.2018.12.009 30622020

[3] Li C., Androulakaki T., Gao A. Y., Yang F., Saikia H., Peters C., and Skantze G., “Effects of posture and embodiment on social distance in human-agent interaction in mixed reality,” in Proceedings of the 18th International Conference on Intelligent Virtual Agents, pp. 191–196, 2018.

[4] Rivu R., Zhou Y., Welsch R., Mäkelä V., and Alt F., “When friends become strangers: Understanding the influence of avatar gender on interpersonal distance in virtual reality,” in IFIP Conference on Human-Computer Interaction, pp. 234–250, 2021.

[5] Zibrek K., Niay B., Olivier A.-H., Hoyet L., Pettré J., and Mcdonnell R., “The effect of gender and attractiveness of motion on proximity in virtual reality,” *ACM Transactions on Applied Perception (TAP)*, vol. 17, no. 4, pp. 1–15, 2020.

[6] Mehta V., “The new proxemics: COVID-19, social distancing, and sociable space,” *Journal of urban design*, vol. 25, no. 6, pp. 669–674, 2020.

[7] Welsch R., Wessels M., Bernhard C., Thönes S., and von Castell C., “Physical distancing and the perception of interpersonal distance in the COVID-19 crisis,” *Scientific reports*, vol. 11, no. 1, pp. 1–9, 2021. 34075094 10.1038/s41598-021-90714-5 PMC8169674

[8] Holt D., Zapetis S., Babadi B., and Tootell R., “Changes in Personal Space During the COVID-19 Pandemic: A Virtual Reality Study,” in NEUROPSYCHOPHARMACOLOGY, pp. 509–510, 2021.33191400

[9] Novick D., and Rodriguez A. E., “A Comparative Study of Conversational Proxemics for Virtual Agents,” in International Conference on Human-Computer Interaction, pp. 96–105, 2021.

[10] Zibrek K., Niay B., Olivier A.-H., Pettré J., Hoyet L., and Mcdonnell R., “Proximity in VR: The Importance of Character Attractiveness and Participant Gender,” in 2022 IEEE Conference on Virtual Reality and 3D User Interfaces Abstracts and Workshops (VRW), pp. 672–673, 2022.

[11] Lisi M. P., Fusaro M., Tieri G., and Aglioti S. M., “Humans adjust virtual comfort-distance towards an artificial agent depending on their sexual orientation and implicit prejudice against gay men,” *Computers in Human Behavior*, vol. 125, pp. 106948, 2021.

[12] Zibrek K., Kokkinara E., and McDonnell R., “Don’t stand so close to me: investigating the effect of control on the appeal of virtual humans using immersion and a proximity-based behavioral task,” in Proceedings of the ACM Symposium on Applied Perception, pp. 1–11, 2017.

[13] Llobera J., Spanlang B., Ruffini G., and Slater M., “Proxemics with multiple dynamic characters in an immersive virtual environment,” *ACM Trans*. *Appl*. *Percept*., vol. 8, no. 1, pp. Article 3, 2010.

[14] Petrak B., Weitz K., Aslan I., and André E., “Let me show you your new home: studying the effect of proxemic-awareness of robots on users’ first impressions,” in 2019 28th IEEE international conference on robot and human interactive communication (RO-MAN), pp. 1–7, 2019.

[15] Petrak B., Stapels J. G., Weitz K., Eyssel F., and André E., “To move or not to move? Social acceptability of robot proxemics behavior depending on user emotion,” in 2021 30th IEEE International Conference on Robot & Human Interactive Communication (RO-MAN), pp. 975–982, 2021.

[16] Bönsch A., Radke S., Ehret J., Habel U., and Kuhlen T. W., “The impact of a virtual agent’s non-verbal emotional expression on a user’s personal space preferences,” in Proceedings of the 20th ACM International Conference on Intelligent Virtual Agents, pp. 1–8, 2020.

[17] Shiomi M., Shatani K., Minato T., and Ishiguro H., “How should a Robot React before People’s Touch?: Modeling a Pre-Touch Reaction Distance for a Robot’s Face,” *IEEE Robotics and Automation Letters*, vol. 3, no. 4, pp. 3773–3780, 2018.

[18] Mejía D. A. C., Saito A., Kimoto M., Iio T., Shimohara K., Sumioka H., et al, “Modeling of Pre-Touch Reaction Distance for Faces in a Virtual Environment,” *Journal of Information Processing*, vol. 29, pp. 657–666, 2021.

[19] Huang A., Knierim P., Chiossi F., Chuang L. L., and Welsch R., “Proxemics for Human-Agent Interaction in Augmented Reality,” in CHI Conference on Human Factors in Computing Systems, pp. 1–13, 2022.

[20] Li R., van Almkerk M., van Waveren S., Carter E., and Leite I., “Comparing human-robot proxemics between virtual reality and the real world,” in 2019 14th ACM/IEEE International Conference on Human-Robot Interaction (HRI), pp. 431–439, 2019.

[21] Kroczek L. O., Böhme S., and Mühlberger A., “Face masks reduce interpersonal distance in virtual reality,” *Scientific reports*, vol. 12, no. 1, pp. 1–10, 2022. 35140279 10.1038/s41598-022-06086-x PMC8828850

[22] Rossi S., Staffa M., Bove L., Capasso R., and Ercolano G., “User’s Personality and Activity Influence on HRI Comfortable Distances,” in International Conference on Social Robotics, pp. 167–177, 2017.

[23] Peeri N. C., Shrestha N., Rahman M. S., Zaki R., Tan Z., Bibi S., et al, “The SARS, MERS and novel coronavirus (COVID-19) epidemics, the newest and biggest global health threats: what lessons have we learned?,” *International journal of epidemiology*, vol. 49, no. 3, pp. 717–726, 2020. doi: 10.1093/ije/dyaa033 32086938 PMC7197734

[24] Pendar M.-R., and Páscoa J. C., “Numerical modeling of the distribution of virus carrying saliva droplets during sneeze and cough,” *Physics of Fluids*, vol. 32, no. 8, pp. 083305, 2020. doi: 10.1063/5.0018432 35002198 PMC8726427

[25] Tamantini C., di Luzio F. S., Cordella F., Pascarella G., Agro F. E., and Zollo L., “A robotic health-care assistant for COVID-19 emergency: A proposed solution for logistics and disinfection in a hospital environment,” *IEEE Robotics & Automation Magazine*, vol. 28, no. 1, pp. 71–81, 2021.

[26] Sumioka H., Yamato N., Shiomi M., and Ishiguro H., “A minimal design of a human infant presence: a case study toward interactive doll therapy for older adults with dementia,” *Frontiers in Robotics and AI*, pp. 164, 2021. 10.3389/frobt.2021.633378 PMC8247474 34222346

[27] Bauer E. A., “Personal space: A study of blacks and whites,” *Sociometry*, pp. 402–408, 1973.

[28] White M. J., “Interpersonal distance as affected by room size, status, and sex,” *The Journal of Social Psychology*, vol. 95, no. 2, pp. 241–249, 1975.

[29] Barrios B. A., Corbitt L. C., Estes J. P., and Topping J. S., “Effect of a social stigma on interpersonal distance,” *The Psychological Record*, vol. 26, no. 3, pp. 343–348, 1976.

[30] Wittig M. A., and Skolnick P., “Status versus warmth as determinants of sex differences in personal space,” *Sex roles*, vol. 4, no. 4, pp. 493–503, 1978.

[31] Long G. T., Selby J. W., and Calhoun L. G., “Effects of situational stress and sex on interpersonal distance preference,” *The Journal of Psychology*, vol. 105, no. 2, pp. 231–237, 1980. doi: 10.1080/00223980.1980.9915156 7400989

[32] Shimizu K., Kihara Y., Itou K., Tai K., and Furuna T., “How perception of personal space influence obstacle avoidance during walking: differences between young and older adults,” *Physical Therapy Research*, vol. 23, no. 1, pp. 31–38, 2020. doi: 10.1298/ptr.E9988 32850276 PMC7344360

[33] Bartneck C., Kulić D., Croft E., and Zoghbi S., “Measurement instruments for the anthropomorphism, animacy, likeability, perceived intelligence, and perceived safety of robots,” *International Journal of Social Robotics*, vol. 1, no. 1, pp. 71–81, 2009.

[34] Rinck M., Rörtgen T., Lange W.-G., Dotsch R., Wigboldus D. H., and Becker E. S., “Social anxiety predicts avoidance behaviour in virtual encounters,” *Cognition and Emotion*, vol. 24, no. 7, pp. 1269–1276, 2010.

[35] Serrurier A., Neuschaefer-Rube C., and Röhrig R., “Past and Trends in Cough Sound Acquisition, Automatic Detection and Automatic Classification: A Comparative Review,” *Sensors*, vol. 22, no. 8, pp. 2896, 2022. doi: 10.3390/s22082896 35458885 PMC9027375

[36] Mouawad P., Dubnov T., and Dubnov S., “Robust detection of COVID-19 in cough sounds,” *SN Computer Science*, vol. 2, no. 1, pp. 1–13, 2021. 10.1007/s42979-020-00422-6 PMC7802616 33458700

[37] Pramono R. X. A., Imtiaz S. A., and Rodriguez-Villegas E., “Automatic cough detection in acoustic signal using spectral features,” in 2019 41st Annual International Conference of the IEEE Engineering in Medicine and Biology Society (EMBC), pp. 7153–7156, 2019. doi: 10.1109/EMBC.2019.8857792 31947484

[38] Hajure M., Tariku M., Bekele F., Abdu Z., Dule A., Mohammedhussein M. et al, “Attitude towards COVID-19 vaccination among healthcare workers: a systematic review,” *Infection and Drug Resistance*, vol. 14, pp. 3883, 2021. doi: 10.2147/IDR.S332792 34584432 PMC8464326

[39] Daoust J.-F., “Elderly people and responses to COVID-19 in 27 Countries,” *PloS one*, vol. 15, no. 7, pp. e0235590, 2020. doi: 10.1371/journal.pone.0235590 32614889 PMC7332014

[40] Walters M. L., Dautenhahn K., Te Boekhorst R., Koay K. L., Syrdal D. S., and Nehaniv C. L., “An empirical framework for human-robot proxemics,” *Procs of new frontiers in human-robot interaction*, 2009.

[41] Bruno N., and Muzzolini M., “Proxemics Revisited: Similar Effects of Arms Length on Men’s and Women’s Personal Distances,” *Universal Journal of Psychology*, vol. 1, no. 2, pp. 46–52, 2013.

[42] Stulp G., Buunk A. P., Verhulst S., and Pollet T. V., “Human height is positively related to interpersonal dominance in dyadic interactions,” *PloS one*, vol. 10, no. 2, pp. e0117860, 2015. doi: 10.1371/journal.pone.0117860 25719490 PMC4342156

[43] Lieberz K. A., Windmann S., Geniole S. N., McCormick C. M., Mueller‐Engelmann M., Gruener F. et al, “The facial width‐to‐height ratio determines interpersonal distance preferences in the observer,” *Aggressive behavior*, vol. 43, no. 5, pp. 460–470, 2017. doi: 10.1002/ab.21704 28261811

[44] Neggers M. M., Cuijpers R. H., Ruijten P. A., and IJsselsteijn W. A., “Determining Shape and Size of Personal Space of a Human when Passed by a Robot,” *International Journal of Social Robotics*, vol. 14, no. 2, pp. 561–572, 2022.

[45] Bailenson J. N., Blascovich J., Beall A. C., and Loomis J. M., “Interpersonal distance in immersive virtual environments,” *Personality and social psychology bulletin*, vol. 29, no. 7, pp. 819–833, 2003. doi: 10.1177/0146167203029007002 15018671

